# *Saccharomyces cerevisiae* cell surface display technology: Strategies for improvement and applications

**DOI:** 10.3389/fbioe.2022.1056804

**Published:** 2022-12-07

**Authors:** Chenmeng Zhang, Hongyu Chen, Yiping Zhu, Yu Zhang, Xun Li, Fei Wang

**Affiliations:** ^1^ Jiangsu Co Innovation Center of Efficient Processing and Utilization of Forest Resources, College of Chemical Engineering, Nanjing Forestry University, Nanjing, China; ^2^ Jiangsu Provincial Key Lab for Chemistry and Utilization of Agro Forest Biomass, Jiangsu Key Lab of Biomass Based Green Fuels and Chemicals, Nanjing, China; ^3^ International Innovation Center for Forest Chemicals and Materials, Nanjing Forestry University, Nanjing, China

**Keywords:** *Saccharomyces cerevisiae*, cell-surface display technology, scaffoldin, bioconversion, cell factory

## Abstract

Microbial cell surface display technology provides a powerful platform for engineering proteins/peptides with enhanced properties. Compared to the classical intracellular and extracellular expression (secretion) systems, this technology avoids enzyme purification, substrate transport processes, and is an effective solution to enzyme instability. *Saccharomyces cerevisiae* is well suited to cell surface display as a common cell factory for the production of various fuels and chemicals, with the advantages of large cell size, being a Generally Regarded As Safe (GRAS) organism, and post-translational processing of secreted proteins. In this review, we describe various strategies for constructing modified *S. cerevisiae* using cell surface display technology and outline various applications of this technology in industrial processes, such as biofuels and chemical products, environmental pollution treatment, and immunization processes. The approaches for enhancing the efficiency of cell surface display are also discussed.

## 1 Background

Cell surface display technology is an attractive method for immobilizing functional proteins/peptides and imparting specific functions to microbial cells. In the cell surface display system, functional proteins/peptides are fused with anchoring protein genes and expressed on the cell surface or plasma membrane under the guidance of signal peptides. The cell-surface display technology has unique advantages over intracellular expression and secretion: 1) Proteins displayed on the cell surface can maintain their functions and properties more stably under harsh temperature or pH conditions than free proteins. 2) The proteins immobilized on the cell surface can be recycled by filtration or centrifugation, avoiding the complicated process of lysing cells or purifying proteins from the cultural liquid. 3) The multi-enzyme cell surface co-display system can shorten the substrate transfer distance, and the co-display of enzymes with synergistic effects can trigger synergistic proximity effects and improve catalytic efficiency. 4) Engineered yeast with functional proteins displayed on the surface can act on large molecular masses of substrates (e.g., cellulose, hemicellulose) that cannot enter the cell, and the resulting monomers (e.g., glucose) can be directly utilized by the cell to produce valuable products (e.g., bioethanol). This process contributes to the integrated bioprocessing process that combines hydrolysis, saccharification and fermentation. Cell surface display systems have many potential applications, including biocatalysts, high-throughput library screening, biological sorbents, oral vaccines, *etc.* (Teymennet-Ramírez et al., 2022).

Cell surface display systems have been successfully applied to various microbial cells. Among them, yeast cells are one of the most suitable host cells for cell surface display, with relatively large cell size, rigid cell walls, and allowing post-translational processing (including folding and glycosylation, *etc.*) of expressed heterologous eukaryotic proteins (Liu et al., 2016a). In yeast, cell surface display was first developed in *S. cerevisiae* with remarkable results. Subsequently, surface display systems using full-length or anchored structural domains of *S. cerevisiae* cell wall proteins were applied to other yeasts, including *Pichia pastoris* (Zhao N. et al., 2017; Yang et al., 2017; Ce et al., 2022) and *Yarrowia lipolytica* (An et al., 2016). *S. cerevisiae* is easy to manipulate genetically and has Generally Regarded As Safe (GRAS) status due to its widespread use in the food industry (Cha et al., 2022). In recent years, various applications of yeast surface display systems have been reported by many researchers, and there are also related review articles outlining strategies for improving yeast surface display systems (Teymennet-Ramírez et al., 2022). However, few have provided detailed overviews for one yeast alone. This paper focuses on the progress of research using *S. cerevisiae* as a cell surface display object and not only introduces strategies to improve cell surface display but also discusses in more detail the wide range of applications of surface-engineered *S. cerevisiae* and provides insights into the challenges of future commercialization.

## 2 Cell surface display systems in *S. cerevisiae*

Cell surface display is a powerful tool for endowing novel functions on *S. cerevisiae* cells by displaying functional proteins/peptides on the cell surface. The cell surface display system has been enriched by the research on cell wall structure of *S. cerevisiae*. Its inner layer consists mainly of β-linked glucans, which are cross-linked with chitin to maintain cell wall strength, and the outer layer consists mainly of mannoproteins, which are covalently linked to the inner layer, and heterologous proteins can be anchored to the mannoprotein layer to determine cell surface properties (Ye et al., 2021). In the cell surface display platform, different enzymes can be located on the same cell surface and the proximity could improve their synergism. This platform simplifies protein purification in some biocatalytic processes and promotes the construction and reuse of whole-cell catalysts.

### 2.1 Direct cell surface display

**FIGURE 1:**
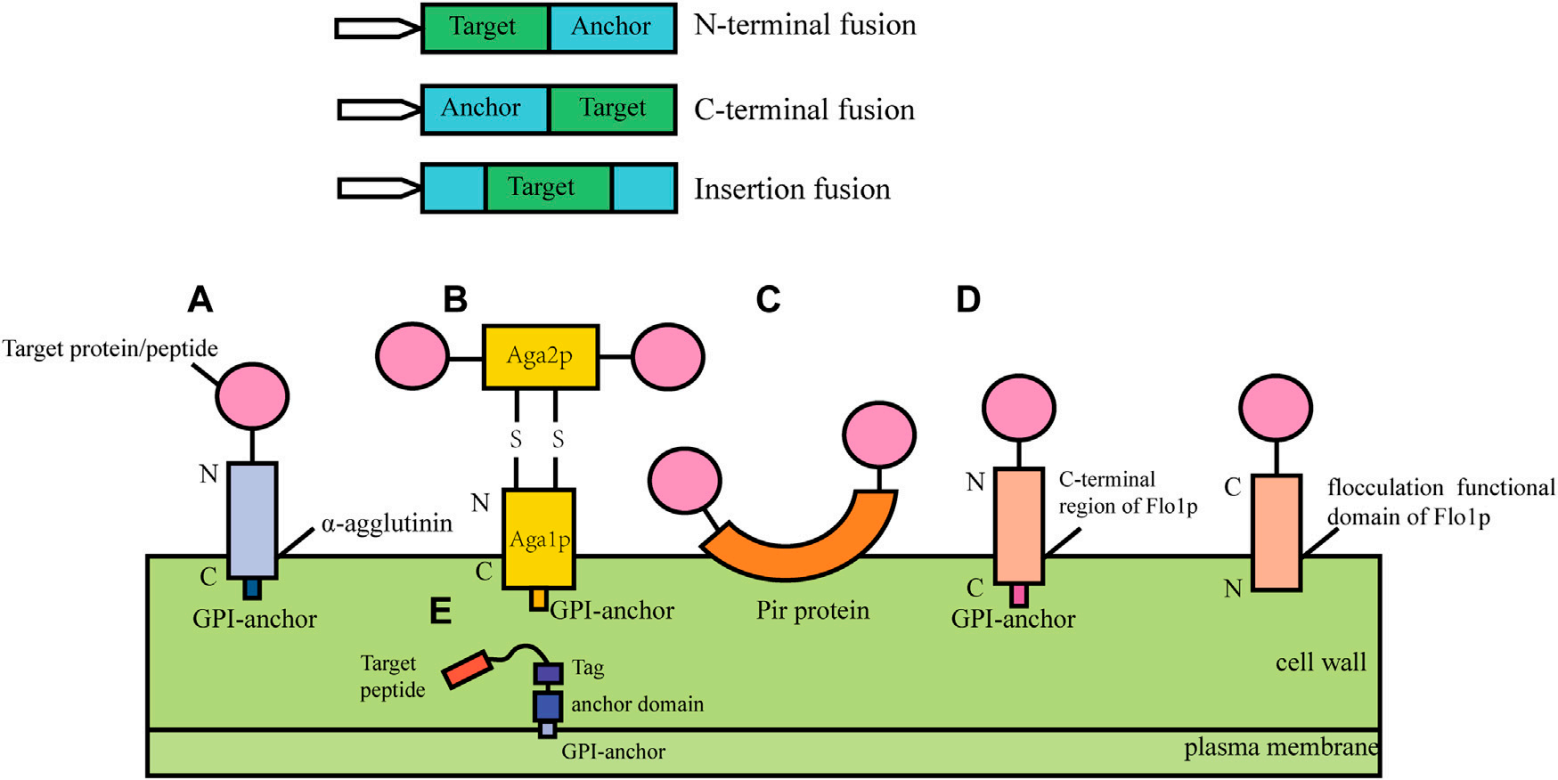
Cell surface display systems in *S. cerevisia.* **(A)** α-agglutinin-based display system; **(B)** a-agglutinin-based display system; **(C)** Pir-based display system; **(D)** Flo1p-based display system; **(E)** Membrane display system.

In cell surface display, the target protein fused with specific vector sequence is introduced into the yeast cell and then the fusion protein is expressed. The signal peptide guides the fusion protein to extracellular secretion, the anchor protein contained in the fusion protein can bind to the cell wall structure of yeast to immobilize the target protein on the surface of the yeast cell (Kondo & Ueda, 2004; Pepper et al., 2008). According to the different fusion modes of anchor proteins, cell surface display systems can be divided into glycosylphosphatidylinositol (Xing et al.) anchor system, FS/FL (truncated forms of Flo1p (Matsumoto et al., 2002), amino acids (FS) 1–1,099 and amino acids 1–1,417) anchor system and Pir protein anchor system (Figure 1). GPI anchor proteins such as Cwp1p, Cwp2p, Tip1p, Flo1p, Sed1p, YCR89w, and Tir1 link to β-1, 6-glucan of the cell wall by the C-terminal GPI-anchor structures, and the target gene is usually fused to the N- terminus (Van der Vaart et al., 1997). Particularly, the a-agglutinin, composed of Aga1 and Aga2, covalently binds to the cell wall by the C-terminal GPI-anchor structure of Aga1, the target protein gene fuses with the C-terminal or N-terminal of Aga2 and Aga2 is connected with Aga1 through a disulfide bond. The FL/FS gene encoding the Flo1p flocculation functional domain is located near the N- terminus of Flo1p and can adhere to mannans in the cell wall by non-covalent interaction, with the target protein generally fused at its C- terminus. Some proteins with active sites near the C-terminus have inhibitory activity when fused with GPI-anchored proteins, and this inhibition can be mitigated using the FL/FS display system (Matsumoto et al., 2002). In addition, the Pir protein display system is suitable for C-terminal fusion, N-terminal fusion and insertion fusion. The protein differs from the first two anchoring modes in that it can form an ester linkage with β-1, 3-glucose in the cell wall *via* N-terminal repeat sequences, or it can attach to specific components of the cell wall *via* disulfide bonds using C-terminal cysteine residues (Yang et al., 2014).

### 2.2 Scaffold-mediated cell surface display

**FIGURE 2:**
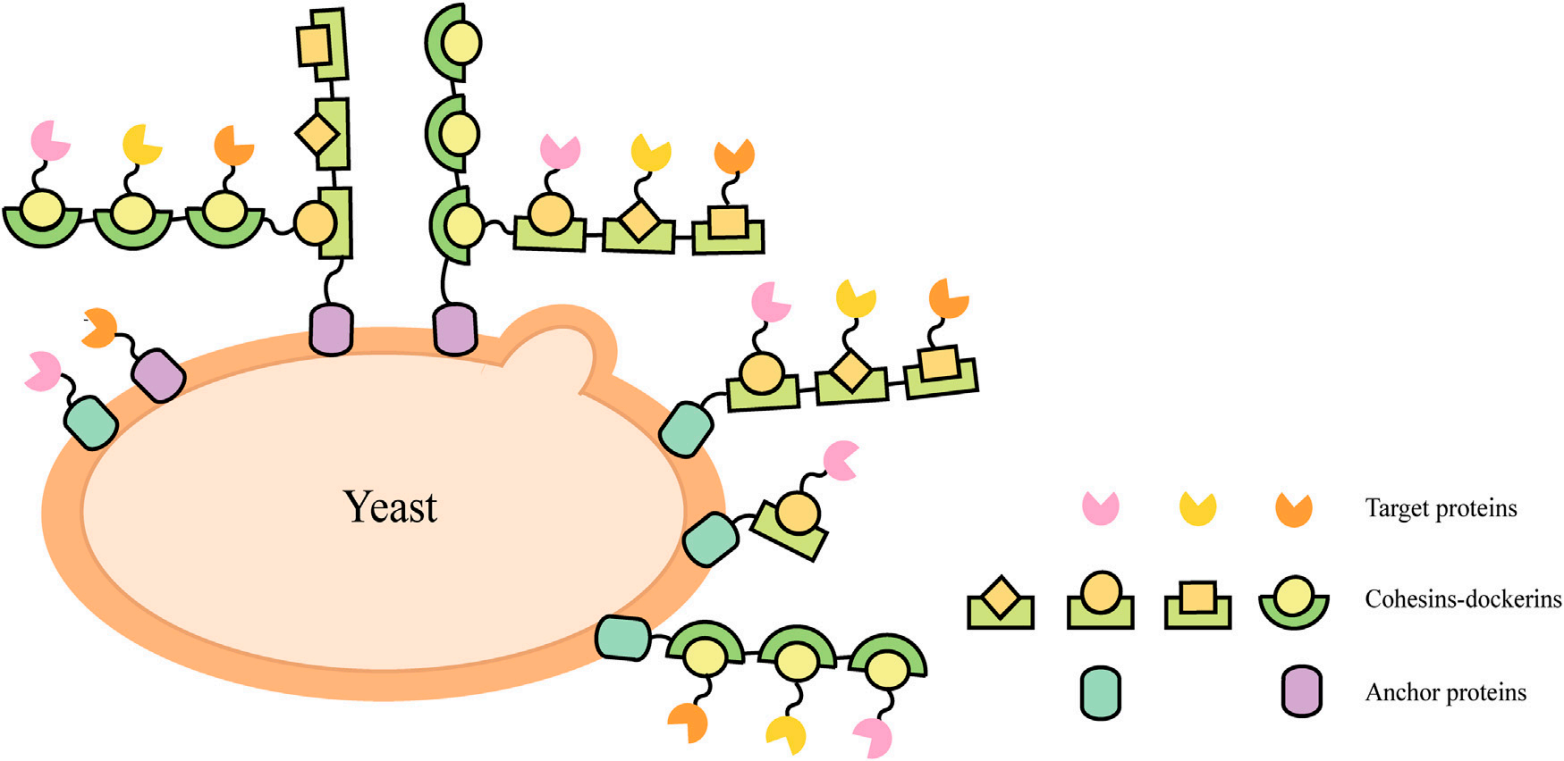
Schematic illustrations of direct and indirect cell-surface display strategies in *S. cerevisiae*.

Natural cellulosomes are often found in anaerobic bacteria (Felix and Ljungdahl, 1993) (such as *Clostridium thermocellum*, *C. cellulolyticum*, *C. cellulovorans*, *Ruminococcus flavefaciens*, *etc.*) and the complex composed of scaffoldins, anchoring domains, cohesins, fiber binding domains, and catalytic units, which provides a novel scaffold-based strategy for cell surface display. This cell-surface display system usually triggers enzyme-enzyme proximity synergistic effects and enzyme-substrate-cell complex synergistic effects. A display method was described to allow the production of all cellulosomal components (miniscaffoldin CipA3 and enzymes) *in vivo*, avoiding the labor-intensive protein purification step (Wen et al., 2010). The complex composition of natural cellulosomes and the considerable molecular weight of scaffolding proteins create a metabolic burden for cell surface display. When multi-enzyme presentation is required, its specificity is not high and the efficiency of self-assembly is reduced. Therefore, the researchers selected cohesin domains and cellulose binding domain (CBD) from different strains to form artificial scaffolds, and selected the anchor proteins from the yeast cell wall as anchor elements to display the scaffolds on the yeast cell surface (Fierobe et al., 2005; Mingardon et al., 2007; Wieczorek and Martin, 2010). Target proteins fused with corresponding dockerins can bind to the scaffold through the interaction between the cohesin and dockerin (Figure 2). Cellulosomal components can be produced within the same cell and assembled on the cell surface, or they can be produced by cells of different strains and then co-incubated to assemble on the cell surface of the displayed scaffold. For example, cells displaying scaffoldins on the surface can be incubated directly with *Escherichia coli* cell lysates containing cellulases to form the cellulosome complex for ethanol production (Tsai S.-L. et al., 2009).

Direct cell surface display is a simple and effective strategy. The enzymes immobilized on the cell surface are stable, and the recycling of the whole cells shortens the purification process of the protein, but it cannot effectively use the cell surface space. When multiple enzymes with synergistic effects are co-displayed, the ratio of various enzymes cannot be well controlled. The scaffold-mediated surface display system can make up for the deficiency of the direct display system, shorten the transfer distance of the substrate and enhance the proximity effect between enzymes. However, the design is more complicated and the assembly stability on the cell surface cannot be guaranteed (Table 1). Therefore, a more appropriate design is needed to construct cell surface display engineering yeasts to make it more practical.

**TABLE 1:** Advantages and disadvantages of each strategy.

Strategies	Advantage	Disadvantages	References
Direct cell surface display	Simple and effective	Not easy to control the ratio when multiple enzymes are co-displayed	Huang et al. (2020)
	High stability	Limited utilization of cell surface space	
	Reusable	Poor site-specificity	
	Easy downstream purification		
Scaffold-mediated cell surface display	Facilitate enzyme cascade reaction	High operational difficulty	Li et al. (2017)
	Improve cell surface space utilization	Complex assembly	
	Can control the proportion of displayed proteins	Poor control of binding stability of complex scaffolds	
	Shorten mass transfer distance	Easy downstream purification	

### 2.3 Improvement of cell-surface display efficiency

The improvement of cell surface display efficiency depends on the effective expression and secretion of target proteins and the activity of the displayed target proteins. To this end, many attempts have been made, such as selecting appropriate anchor proteins and expression vectors, constructing highly expressed gene cassettes, strengthening protein folding and post-translational modification, so as to improve the production and activity of proteins. However, further efforts are needed to achieve universal commercialization.

#### 2.3.1 The important role and localization effect of anchoring domains

The display efficiency of enzymes on the cell surface is related to the anchoring proteins attached (Table 2). Attempts have been made to improve cell surface display efficiency by screening for contrasting new anchoring proteins. Phienluphon et al. (2019) selected 37 GPI-type anchor proteins from different sources by computational prediction, fused with yEGFP, pre-screened by comparing the fluorescence intensity, and further fused several vigorous fluorescence anchor proteins with β-glucosidase to determine the enzymatic activity. Finally, 6_Kl from *Kluyveromyces lactis* was found to have higher transcript levels and superior target protein display efficiency. By comparing the display efficiency and enzyme activity of several display systems, Yang et al. (2019) adapted the a-agglutinin anchoring system to develop a new surface display system by using α-galactosidase as the target enzyme and fusing it directly to Aga1p. The enzyme was also fused to conventional a-agglutinin and to six other anchoring proteins selected. A comparison of enzyme activity revealed that Aga1p, Dan4p, and Sed1p had high display efficiency and were promising anchoring systems for immobilizing recombinant proteins on the yeast surface. The display efficiency of the anchor protein domain varies with the molecular weight of the displayed protein. The localization effect of structural anchoring domains may also affect the display efficiency of target proteins on the cell surface. Inokuma et al. (2020) fused the anchored domains (Sed1p and Sag1p) with heterologous proteins and expressed them in *S. cerevisiae.* It was observed by fluorescence and immunoelectron microscopy that the two domains had different anchorings on the cell surface, with sed1p-anchoring domain mainly localized on the outer surface of the cell wall, while sag1p-anchoring domain was mainly localized inside the cell wall. The cell wall space can be effectively utilized by properly controlling the anchoring position, making the cell surface display technology more useful in various fields.

**TABLE 2:** Comparison of anchoring efficiencies of different anchor proteins.

Enzyme origin	Anchor protein	Yeast host	Promoter	Activity	References
β-glucosidase (BGL1) from *Aspergillus aculeatus*	SAG1	*S. cerevisiae*	SED1	99 ± 10 U/g cell dry weight	Inokuma et al. (2014)
	SED1	*S. cerevisiae*	SED1	235 ± 28 U/g cell dry weight	Inokuma et al. (2014)
BGL1 from *Saccharomycopsis fbuligera*	DAN4	*S. cerevisiae*	TEF1	∼785 U/g cell dry weight	Yang et al. (2019)
	AGA1	*S. cerevisiae*	TEF1	∼920 U/g cell dry weight	Yang et al. (2019)
BGL from *Aspergillus niger*	Sag1	*S. cerevisiae*	GPD	∼18 U/g cell dry weight	Zhang et al. (2019)
	Sed1	*S. cerevisiae*	GPD	25.22 ± 0.81 U/g cell dry weight	Zhang et al. (2019)
Lipase Lip2 from *Y. lipolytica*	Cwp2	*S. cerevisiae*	GPD	∼8 U/g cell dry weight	Zhang et al. (2019)
	Cwp2	*S. cerevisiae*	PGK	7.6 ± 0.4 U/cell dry weight	Liu et al. (2010)
Lip7 from *Y. lipolytica*	a-agglutinin	*S. cerevisiae*	GAL1	283 U/g cell dry weight	Liu et al. (2010)
	Flo1	*P. pastoris*	AOX1	85 U/g cell dry weight	Jiang et al. (2007)
*Rnizopus oryzae* Lipase (ROL)	FLO1	*S. cerevisiae*	TRP1	61.3 IU/g cell dry weight	Matsumoto et al. (2002)
β-Glucuronidase from *Aspergillus oryzae*	Agα1	*P. pastoris*	GAP	24.32 U/g dry cell weight	Wang et al. (2019)
	Pir1	*P. pastoris*	GAP	28.89 U/g dry cell weight	Wang et al. (2019)

#### 2.3.2 Improvement of protein secretion

Protein secretion is an essential factor in improving the efficiency of cell surface display. The main strategies to improve protein secretion include increasing protein expression levels and signal peptide engineering. The strength of the promoters often determines the expression level of heterologous proteins in *S. cerevisiae*. Different types of promoters differ in their strength. In addition, the strength of promoters can be influenced by other factors such as growth conditions (Sun et al., 2012a) (e.g., glucose concentration), introns (Hoshida et al., 2017) and other factors. Synthetic promoters were constructed using promoter engineering to further improve protein expression levels. The upstream activating sequences (UASs) of different promoters were combined with core promoters to construct a synthetic promoter library. Using this approach, galactose-inducible promoters stronger than P_GAL1_ were successfully constructed by fusing UAS_GAL1_ to the core promoters of TDH3 and TEF1 (Deng et al., 2021). The selection of efficient signal peptides helps to enhance protein secretion and increase the number of proteins displayed on the cell surface. A signal peptide from *S. cerevisiae* SED1 was shown to be an efficiently secreted *A. aculeatus* β-glucosidase (BGL1). The extracellular activity of BGL1 was 1.3-fold and 1.9-fold higher than that of the *Rhizopus oryzae* glucoamylase (GLUASP) and *S. cerevisiae* α-mating pheromone (MFα1SP), respectively (Inokuma et al., 2016). The design mutations of the a-factor preproleader of the MFα1SP have the opportunity to obtain enhanced signal peptides for the secretion of a variety of fungal enzymes (Aza et al., 2021). In addition, some researchers proposed that the fusion of the target protein with a secretion-enhancing peptide cassette and rational optimization of the original peptide could enhance the secretion of the heterologous proteins (Cho et al., 2022). The expression of the enzyme can be further improved by optimizing the combination of promoter and signal peptide. Different promoters and signal peptides constituting nine gene cassettes were selected for fusion expression with *Kluyveromyces marxianus* inulinase gene. The inulinase activity of *S. cerevisiae* carrying the PGK1 promoter and MFα1 signal sequence was the highest (Hong et al., 2015). Terminators affect gene expression by terminating transcription and controlling the half-life of mRNA. Synthetic terminators are short in sequence, easy to synthesize, and more functional. A panel of short (35–70 bp) synthetic terminators was reported to increase fluorescent protein output by 3.7-fold and transcript levels by 4.4-fold compared to the commonly used CYC1 terminator (Curran et al., 2015). The synthetic terminators might function better in *S. cerevisiae* compared to a native promoter.

Other strategies to improve protein secretion include: 1) Overexpression of related genes involved in protein transport or cell surface display (Wentz & Shusta, 2007; Hou et al., 2012; Van Zyl et al., 2016; Bao et al., 2017; Li et al., 2022) and combinatorial regulation of gene expression (Yang et al., 2022). 2) Point mutations and gene deletions (Matsuoka et al., 2014). Point mutation target genes identified from UV-mutagenized strains were analyzed to be involved in multiple intracellular biological processes. Detecting the effects of point mutations and gene deletions on the secretion of α-amylase could help to balance these biological processes and thus improve protein secretion (Wang et al., 2022). 3) Optimization of secretion pathways (Tang et al., 2017; Huang et al., 2018; Besada-Lombana & Da Silva, 2019) and stress modulation (Lamour et al., 2019). 4) Co-overexpression of chaperones (Bae et al., 2016; Bae et al., 2022). 5) The choice of integration method. Auxotrophic integration, cocktail δ-integration (Yamada et al., 2010), CRISPR-δ-integration (Sasaki et al., 2019), and other integration methods (Zhang et al., 2021; Zheng et al., 2022) have been developed successively to increase the copy number of integrated genes, thereby enhancing protein secretory overexpression.

#### 2.3.3 Reduction of steric hindrance and optimization of space utilization on the cell surface of *S. cerevisiae*

The activity of the target protein can be limited by the competition with other GPI proteins for the limited binding sites on the cell wall when displayed on the cell surface (Van der Vaart et al., 1997). For example, the β-Glucosidase activity of the SED1 disruptant was 22% higher than that of the Sed1-undisrupted strain (Bamba et al., 2018), the disruption of CWP2 and YGP1 could increase BGL activity by 63% and 24%, respectively, compared with the original strain (Arnthong et al., 2022), and CCW12 and CCW14 co-knockout resulted in increased cell wall thickness, which may increase the number of heterologous proteins displayed on the cell surface (Inokuma et al., 2021).

The addition of a suitable length of linker peptide between the anchor and target proteins seems to help to separate the active part of the protein from the cell wall, creating space for substrate access and improving the activity of the display enzyme (Washida et al., 2001; Ullah et al., 2017; Yang et al., 2019).

**FIGURE 3:**
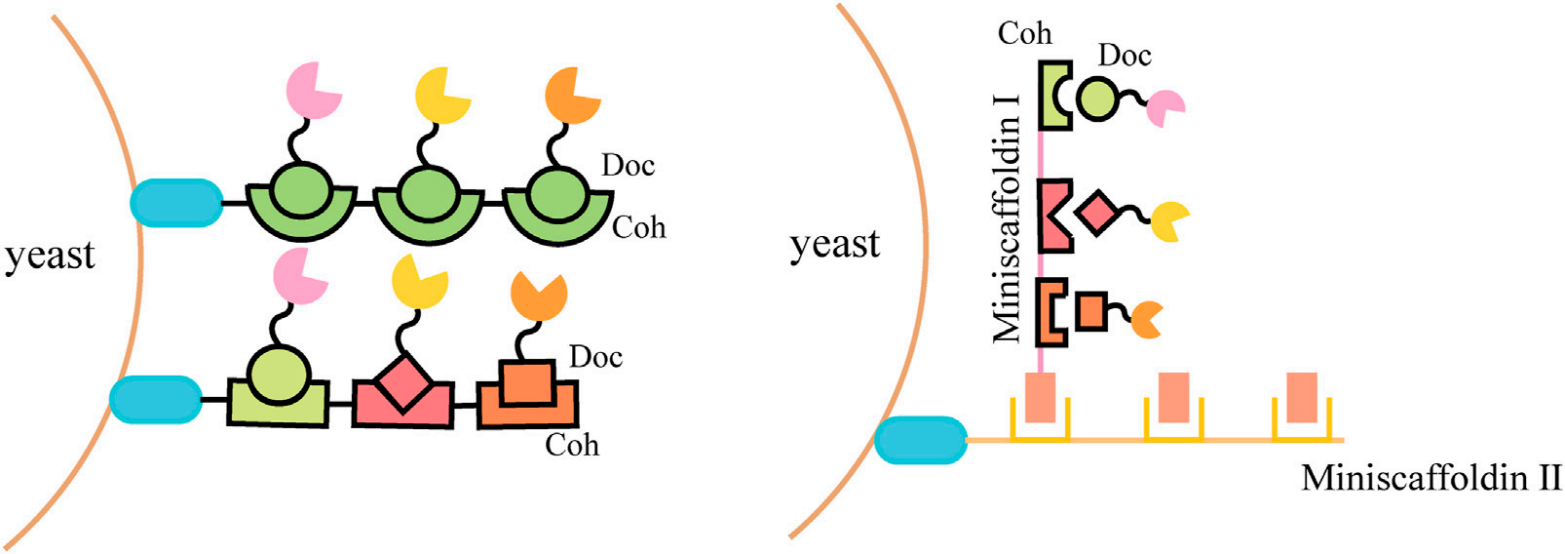
Schematic diagram of the assembly strategy on the yeast cell surface using scaffoldin.

To further improve the space utilization and display efficiency of the cell surface, the introduction of scaffoldin is a potential strategy. Scaffoldin could be enhanced by increasing cohesion number and utilizing double-layered scaffoldins (Figure 3). The *ex vivo* assembly of a functional tetravalent designer cellulosome on the yeast cell surface was developed and exhibited a 4.2-fold enhancement in the hydrolysis of phosphoric acid swollen cellulose (PASC) compared with free enzymes (Tsai et al., 2013). Combining a two-scaffoldin-based cellulosome with an intracellular cellodextrin pathway in *S. cerevisiae* enabled the co-utilization of cellulose-mixed sugars (Fan et al., 2016; Li et al., 2017). A synthetic scaffoldin ScafAGA3 was constructed using the repeated N-terminus of Aga1p. In addition, the scaffoldin ScafCipA3 from *C. thermocellum* fused with Aga2p was attached to the scaffoldin ScafAGA3 *via* disulfide bonds, and the secreted cellulases assembled to ScafCipA3, allowing the formation of a complex cellulosome with two scaffoldins. The newly designed cellulosomes enabled yeast to ferment cellulose directly to ethanol (1.52 g/L) by regulating the ratio of scaffoldins and cellulases (Tang et al., 2018).

## 3 Applications of cell-surface display engineered *S. cerevisiae*

**FIGURE 4:**
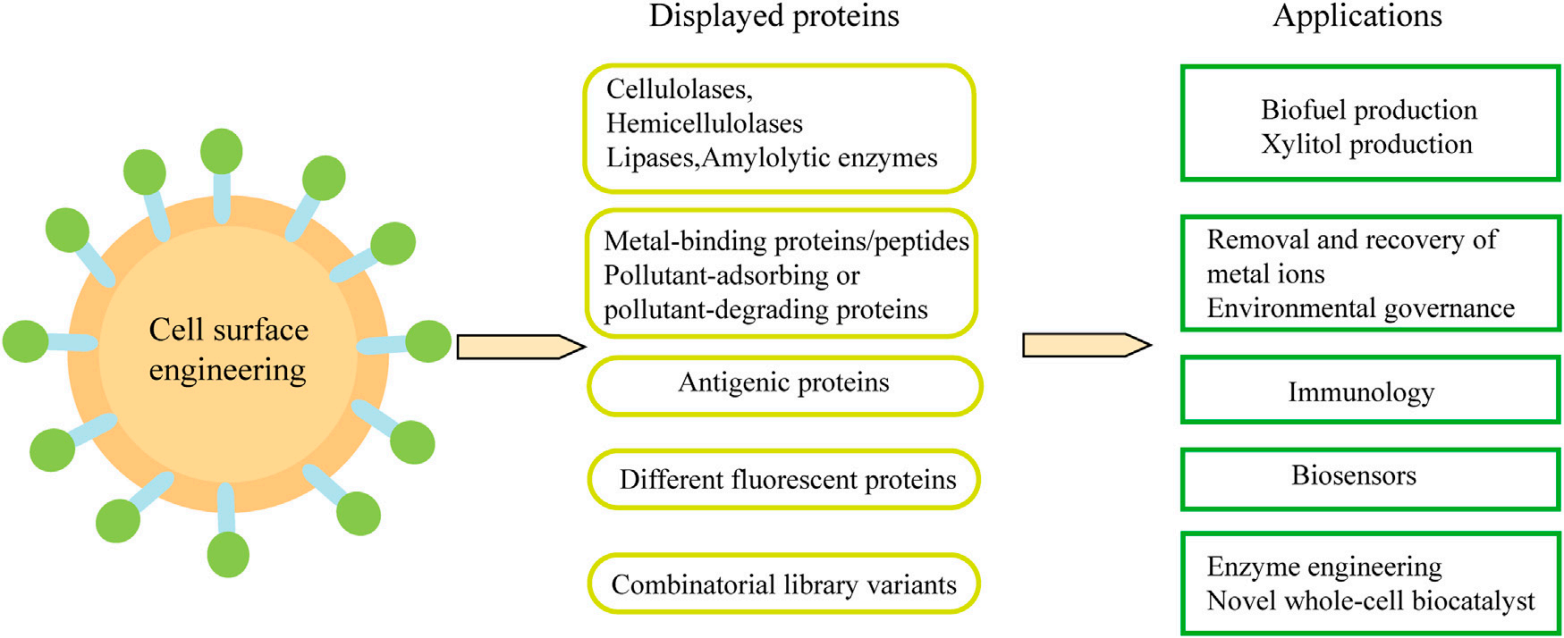
Applications of *S. cereviaiae* cell surface engineering.

Cell-surface display technology can be used to produce biofuels and various chemicals from cheap sources rich in sugars, triglycerides and waste protein. In addition, it is also widely used in biological adsorbents and oral vaccines. Combined with metabolic engineering and synthetic biology methods, engineered yeast cells with desired functions can be constructed (Figure 4).

### 3.1 Applications in bioconversion of agricultural and forestry waste

Agricultural and forestry wastes are produced in large quantities worldwide, resulting in a certain amount of resource waste and environmental problems. From the perspective of the circular economy, the central task is to refine agricultural and forestry wastes to produce valuable energy and chemicals (Song et al., 2020). A variety of enzymes including cellulase, hemicellulases, lipase, amylase, cyclodextrin glucan invertase, amylase and other enzymes have been successfully expressed on the surface of yeast cells with biological activity. The successfully constructed engineered strains can not only effectively degrade agroforestry waste rich in lignocellulose and starch, but also lay the foundation for the simultaneous saccharification and fermentation (SSF)/consolidation bioprocessing (CBP) process. ‬‬‬‬‬‬‬‬‬‬‬‬‬‬‬‬‬‬‬‬

#### 3.1.1 Bioconversion of cellulose catalyzed by engineered *S. cerevisiae*

Cellulose, composed of glucose connected by β-1, 4-glycosidic bonds, is the most abundant macromolecular polysaccharide in nature. At least three cellulases are required to hydrolyze cellulose into glucose: endoglucanase (EG), cellobiohydrolase (CBH) and β-glucosidase (BGL). EG randomly hydrolyzes the β-1,4-glycosidic bonds by acting on amorphous regions of cellulose, and shortens the long-chain cellulose molecules to produce a larger number of shorter cellulose chains with reducing and non-reducing ends; CBH cuts off cellobiose units from the reducing/non-reducing ends of cellulose, and acts mainly on the crystalline region of cellulose (Hu et al., 2015); BGL hydrolyzes cellobiose into glucose. These three enzymes have been successfully displayed on the cell surface of *S. cerevisiae* to hydrolyze cellulose to glucose (Table 3). Placing EGII from *Trichoderma reesei* and CBHI from *Talaromyces emersonii* in the same space (on the cell surface or in the medium) facilitates the fermentation of amorphous cellulose-based ethanol (Liu et al., 2015). Engineering *S. cerevisia* demonstrated that these two enzymes, along with two others (CBH2 from *Chrysosporium lucknowense* and BGL from *Aspergillus aculeatus*) acted on amorphous cellulose and crystalline cellulose, exhibiting higher ethanol yields because CBH2 reduced the bumpy surface of the cellulose and facilitated the movement of CBH1 by enhanced synergistic hydrolysis (Liu et al., 2016b). Many attempts have been made to improve the fermentation process and introduce new lignocellulose-degrading enzymes (e.g., hydrolytic polysaccharide monooxygenases (LPMOs) and cellobiose dehydrogenases (CDHs)) to further enhance the degradation and product yield of cellulose (Quinlan et al., 2011; Horn et al., 2012; Matano et al., 2012; Nakatani et al., 2013; Bae et al., 2015; Cunha et al., 2021).

**TABLE 3:** Applications of *S. cerevisiae* cell surface display in cellulose degradation.

Strains	Ethanol yield	Substrate	Enzymes	Anchor proteins	References
*S. cerevisiae* BY4741	EG-D-CBHI-D, 2.9 g/L; EG-S-CBHI-S, 2.6 g/L	10 g/L PASC	EG II, CBH I, BGL	Sed1	Liu et al. (2015)
*S. cerevisiae* BY4741	1.3 g/L	100 g/L MC6	EG, BGL, CBHI, CBHII	Sed1	Liu et al. (2016b)
*S. cerevisiae* MT8-1	3.4 g/L	20 g/L PASC	SWOI, EGII, CBHII, BGL1	α-agglutinin	Nakatani et al. (2013)
*S. cerevisiae* NBRC1440DHUWL	39.4 ± 2.3 g/L	200 g-DW/L cellulosic material	BGL1, CBH2, EG2	a-agglutinin	Matano et al. (2012)
*S. cerevisiae* CEN.PK102-5B	1.52 g/L	PASC	CBHI, CelA, BGL1	Aga1-Aga2	Tang et al. (2018)
*S. cerevisiae* EBY100	1.80 g/L	PASC	CelA, CBHII, BGLI	Aga1-Aga2	Kim et al. (2013)
*S. cerevisiae* EBY100	4.3 g/L	10 g/L CMC and batch fed sucrose	celcca (EG), CA_C0911 (CBH)	Aga1-Aga2	Li et al. (2017)
	2.9 g/L	xylose and 10 g/L CMC			
	1.2 g/L	xylose and 10 g/L PASC			
*S. cerevisiae* EBY100	1.8 g/L 94 h	PASC	CBHII, BGL1, CelA	Aga1-Aga2	Kim et al. (2013)
*S. cerevisiae* EBY100	2.7 g/L	PASC	GH61a (a LPMO), CDH, EGII, CBHII, BGL1	Aga1-Aga2	Liang et al. (2014)
*S. cerevisiae* EBY100	1,412 mg/L	Avicel	celCCA (EG), celCCE (CBH), Ccel_2454 (BGL)	Aga1-Aga2	Fan et al. (2012)
*S. cerevisiae* EBY100	1,138 mg/L	Avicel	celCCA, celCCE, Ccel_2454	Aga1-Aga2	Fan et al. (2013)
	1086 mg/L	PASC			
*S. cerevisiae* EBY100	8.61 g/L	20 g/L galactose and 10 g/L CMC	celcca, CA_C0911	Aga1-Aga2	Fan et al. (2016)
	9.97 g/L	20 g/L galactose and 10 g/L PASC			
*S. cerevisiae* L2612	0.95 g/L	10 g/L birchwood xylan	XynII, AbfB XlnD	Aga1-Aga2	Sun et al. (2012b)
*S. cerevisiae* BY4741	2.9 g/L	10 g/L Avicel	EG2, CBH1, CBH2, BGL	Sed1	Liu et al. (2017)
Commercial ethanol *S.cerevisiae*	(>50 g/L)	cheese whey and pretreated corn cob	BGL1, EG, CBH1, CBH2	Sed1 SAG1	Cunha et al. (2021)

*S. cerevisiae*, *Saccharomyces cerevisiae*. BGL, β-glucosidase; EG, CelA, endoglucanase; CBH, cellobiohydrolase; SWO I, expansin-like protein; LMPO, lytic polysaccharide monooxygenase; CDH, cellobiose dehydrogenase; xylosidase, Xln; endoxylanase, Xyn; α-L-arabinofuranosidases, AbfB.

The development of multiple scaffoldins (e.g., CipA, ZZ-Coh-Coh, and ScafAGA3) can help to control the ratio of cell surface display proteins and improve ethanol production more effectively (Dong et al., 2020; Qi et al., 2021). A pentafunctional minicellulosome composed of LPMOs, CDHs, CBH, EG and BGL was generated and grown with phosphoric acid swollen cellulose as the sole carbon source (Liang et al., 2014). The delicate balance between the oxidative activity and classical hydrolyase is of importance for the degradation of cellulosic materials (Cannella and Jørgensen, 2014). Expression of all cellulosome components in a single strain may cause heavy metabolic burden and blockage of potential secretion mechanism, thus reducing enzyme activity (Wen et al., 2010). Therefore, displaying anchoring scaffoldins and secreting catalytic and non-catalytic units should be considered to be divided into two independent steps, which can decrease the metabolic burden of the yeast host (Tsai S. L. et al., 2009; Goyal et al., 2011). Researchers achieved an enhancement in the yield by increasing the complexity of the scaffold, that is, by constructing the cellulosome construction and its attachment by two individual miniscaffoldins. The engineered *S. cerevisae* EBY100 could directly convert avicel to bioethanol (1,412 mg/L) (Fan et al., 2012). Others constructed novel cellulolytic yeast consortiums for cellulosic ethanol production (yield up to 1.87 g/L) (Tsai et al., 2010; Kim et al., 2013). The major drawbacks of hydrolyzing cellulose into glucose before cellular uptake are: 1) Inefficient cellulose hydrolysis caused by glucose inhibition on extracellular cellulases. 2) Glucose-induced carbon catabolite repression affects the co-utilization of other sugars. Reconstitution of the cellodextrin transport system in *S. cerevisiae* has been reported (Galazka Jonathan et al., 2010; Choi et al., 2022; Ylinen et al., 2022). This system promotes the efficient utilization of cellulose and mitigates the inhibitory effect of glucose (Fan et al., 2016; Li et al., 2017).

#### 3.1.2 Bioconversion of hemicellulose catalyzed by engineered *S. cerevisiae*

Xylan, the main component of hemicellulose is the second-most polysaccharide in lignocellulosic materials. It is more easily degraded into monomers than cellulose through pretreatment processes, making it an attractive source of sugar for bioethanol fermentation. With the gradual maturity of cell surface display engineering and the heterologous expression of xylose metabolizing enzymes, a one-step process for xylan assimilation can be realized by engineered *S. cerevisiae*. Xylose-utilizing *S. cerevisiae* can be constructed by introducing the genes encoding enzymes related to the xylose assimilation pathway (Katahira et al., 2006). A series of multifunctional minihemicellulosomes were constructed for the hydrolysis of arabinoxylans. The D-xylose utilization pathway consisting of xylose reductase (XR), xylitol dehydrogenase (XDH), and D-xylose kinase (XK) from *Scheffersomyces stipitis* was integrated into the genome of *S. cerevisiae* L2612, combined with the display of bifunctional minihemicellulosome. Engineering *S. cerevisiae* could convert birchwood xylan directly to ethanol (Sun et al., 2012b). A strategy was proposed to efficiently degrade xylan for ethanol production by controlling the mixed cultures of xylose-utilizing engineered yeast that displaying different hemicellulases (Tabañag et al., 2018). Industrial *S. cerevisiae* strains is considered to be a powerful host for displaying hemicellulose hydrolase on the cell surface and optimizing xylose assimilation because of its good heat resistance and high resistance to inhibitors. The use of industrial strains as gene recombination hosts may be more beneficial for the commercial production of ethanol. By introducing xylan-degrading enzymes and expressed xylose-assimilating enzymes into the recombinant yeast, hemicellulose substrates can be directly degraded to produce a variety of high-value-added products other than ethanol (Cunha et al., 2020).

Xylitol, a high-value-added pentose alcohol, can be produced by one-step reduction of xylose. Industrial production of xylitol from purified D-xylose requires an expensive catalytic hydrogenation process. A recombinant xylose-utilizing *S. cerevisiae* was constructed to directly degrade hemicellulose in rice straw hydrolysate to produce xylitol. The cell surface display technology combined with the incorporation of membrane separation technology further improved xylitol production, with a twofold increase in xylitol production by the multi-enzyme co-display engineered *S. cerevisiae* from the membrane separated hydrolysate (compared to the hydrolysate without membrane separation) (Guirimand et al., 2016). An engineered *S. cerevisiae* expressing cytosolic xylose reductase with β-D-glucosidase, xylosidase and xylanase co-displayed on the cell surface was constructed to produce xylitol from woody Kraft pulp (KP) (Guirimand et al., 2019). These results set the stage for large-scale xylitol production from lignocellulose.

#### 3.1.3 Bioconversion of starch catalyzed by engineered *S. cerevisiae*

Starch, a polymer of α-D-glucose, is generally more easily degraded than cellulose and found in large quantities in many agricultural and industrial wastes. Using surface display engineering *S. cerevisiae* to degrade starch raw materials can not only produce biofuel but also improve the process quality of the food industry. Alpha-amylase and glucoamylase codisplayed *S. cerevisiae* was constructed to produce ethanol directly from corn starch with a yield of 86.5% of the theoretical value (Yamada et al., 2011). The activity of α-amylase was dependent on the anchor protein, and the activity of the strains based on flocculin system was 40 times higher than that based on agglutinin (Shigechi et al., 2002). Most of the insoluble starch is partially non-degradable (Shigechi et al., 2004), for which some attempts such as increasing enzyme activity to improve starch utilization efficiency have been reported. A diploid yeast strain displaying structurally optimized α-amylase and glucoamylase was successfully constructed. The modified yeast was used to ferment 100 g/L of raw starch with an average ethanol yield of up to 1.61 g/L/h over 23 successive cycles, yielding 76.6% of the theoretical value (Yamakawa et al., 2012). Cyclodextrin glucan transferase (CGTase) was demonstrated on *S. cerevisiae* cell surface to effectively hydrolyze starch to produce glucose and maltose for yeast fermentation and to enhance the bread-baking process (Shim et al., 2007).

#### 3.1.4 Bioconversion of waste oil catalyzed by engineered *S. cerevisiae*

Lipases catalyze a variety of reactions and are widely used in industry, so the cell surface display of lipases is a potential way to construct whole cell catalysts. Biodiesel is a clean and renewable fuel that can be produced from waste rich in triglycerides. The production costs can be reduced by using engineered yeast cells that display lipases on the cell surface as recyclable biocatalysts. Through the flocculation function of Flo1p, the lipase ROL can be displayed on the cell surface of *S. cerevisae*, and the yield of synthesized methylesters from triglyceride and methanol reached 78.3% after 72 h of reaction (Matsumoto et al., 2002). Later *Candida antarctica* lipase B (CALB) and *Yarrowia lipolytica* lipases were also displayed on *S. cerevisae* by α-agglutinin- or Flo1p-display systems (Kato et al., 2007; Liu et al., 2010). *Pichia pastoris* can use methanol as the sole carbon source, and its promoter AOX1 is regulated by methanol and corresponding integrated vectors. The transformants have strong genetic stability and few native proteins are expressed extracellularly. *P. pastoris* lacks α-1, 3-mannosyltransferase, so lipase activity is not affected by excessive glycosylation. Compared with *S. cerevisiae*, *P. pastoris* displaying lipases on the cell surface is more suitable for the high-density culture and generally produce enzyme in higher yield. With the ability to utilize a variety of hydrophobic substrates as carbon sources, high resistance to adversity, and tolerance to high salinity and low temperatures, *Y. lipolytica* is an attractive host for cell surface display (Yuzbasheva et al., 2015; Qiao et al., 2018; Yang et al., 2020).

### 3.2 Applications in environmental control

Heavy metals are frequently detected in industrial wastewater, which are usually not biodegradable and can only be detoxified by means of absorption, enrichment and removal from wastewater (Fu and Wang, 2011). The recovery of rare metals can save resources and reduce environmental pollution. The cell wall of *S. cerevisiae* is mainly composed of mannoproteins, chitin, β-1, 6-glucan and β-1, 3- glucan (Kollár et al., 1995). These biomolecules consist of carboxylate, phosphate, hydroxyl group, amine, sulfhydryl group and other functional groups, which provide a range of different metal binding sites. Different metal-binding peptides combined with anchor systems can be displayed on the cell surface of *S. cerevisiae.* These engineered strains could recover metals from waste solutions and become useful tools for bioreremediation and bioadsorption of environmental pollutants (Mashangoane and Chirwa, 2022). Four types of *Solanum nigrum* metallothionein (SMT) were displayed on *S. cerevisiae* cell surface by using an α-agglutinin-based display system to adsorb ultra-trace cadmium effectively (Qinguo et al., 2016). Similarly, by displaying MerR on the cell surface, the adsorption capacity of *S. cerevisiae* to Hg^2+^ was much higher than that of the original and the control strains, while the engineered yeast strain also exhibited higher tolerance to Hg^2+^ (Wei et al., 2018). In addition to heavy metals, there are many emerging environmental pollutants such as parabens with disruptive effects and ethyl carbamate (EC), a possible human carcinogen. The removal of parabens could be mediated by *Fusarium solani pisi* cutinase (FsC) expressed on the cell surface of *S. cerevisiae* (Zhu and Wei, 2019). The EC degradation could be improved by displaying urethanease (UreA) from *Micrococcus species* on the cell surface of *S. cerevisiae* (Han et al., 2022).

### 3.3 Applications in immunology

In view of the safety of *S. cerevisiae,* it is an excellent vector for vaccine production. An efficient antibody response can be obtained by displaying antigenic proteins on the yeast cell surface and then feeding the vaccine to animals. The antibody response can be enhanced by increasing the number of antigenic proteins displayed on the cell surface. Using the single-chain variable fragment (scFv) of the anti-infectious hematopoietic necrosis virus isolate Sn1203 antibody as a model protein, the a-agglutinin system was used to display *E. coli*-derived and yeast-derived ScFV on the cell surface of *S. cerevisiae*, which effectively increased the number of proteins displayed on the cell surface and enhanced the possibility of the engineered yeast as an oral vaccine (Zhao J.-Z. et al., 2017). VP28 is an envelope protein of White Spot Syndrome Virus (WSSV), which induces a high immune response in shrimp. Probiotics combined with VP28 anchored yeast cell extract could be used as an oral vaccine in shrimp aquaculture (Le Linh et al., 2021). VP24 is another important envelope protein of WSSV. When applied as an oral vaccine, the protective effect of *S. cerevisiae* with VP28 and VP24 fusion protein on cell surface against WSSV attack was more significant than that of with VP28 only, and the survival rate was up to 100% (Lei et al., 2021a). Vaccines made from recombinant viral proteins displayed on the surface of *S. cerevisiae* cells can be more easily recognized by the host mucosal immune system through oral administration, thus triggering protective immunity. This method is not only easy to operate and safe, but also has a short production cycle, which can be used to treat poultry in a short time. EBY100/PYD1-HA was developed as an oral vaccine against avian influenza A (H5N1) infected chickens (Lei et al., 2021b). Timely vaccination is the most effective solution to the outbreak epidemics. An engineered *S. cerevisiae* displaying the receptor binding domains (RBDs) of the spike protein of the initial strain of SARS-CoV-2 and its variants was constructed as a vaccine candidate against the coronavirus (COVID-19) (Xing et al., 2022). In view of the clear genetic background and easy genetic modification of *S. cerevisiae*, the vaccines constructed based on *S. cerevisiae* cell surface display are expected to achieve large-scale production to prevent various infectious diseases.

### 3.4 Applications in other areas

Cell surface engineering yeast can also be used in biosensors, protein library screening and other fields (Han et al., 2018; Zhao et al., 2021). Cell surface display of fluorescent proteins exerts less metabolic stress on cells than intracellular expression. Displaying a visible reporter on the cell surface may help characterize cells at the single-cell level. Under the control of different promoters, different fluorescent protein variants were displayed on the surface of *S. cerevisiae*, and the engineered yeast could be used as a reporting system to monitor environmental changes and the production of foreign proteins. The GFP cell surface display gene cassette controlled by GAPDH promoter and the BFP cell surface display gene cassette controlled by UPR-ICL promoter were both integrated into the genome of *S. cerevisiae*. The GAPDH promoter acted when the glucose concentration was high in the early stage of culture, and GFP was displayed on the cell surface emitting green fluorescence. When the glucose concentration was low, the UPR-ICL promoter acted and BFP was co-displayed on the cell surface emitting blue fluorescence. The fluorescence intensity and color on the cell surface changed with the intracellular and extracellular glucose concentrations, so the surface display system controlled by the promoters could be used to monitor the glucose concentration inside and outside the cell (Shibasaki et al., 2001). Furthermore, a system was constructed to monitor the production of foreign proteins in yeast by the same promoter controlling the production of foreign proteins and the surface display of reporter gene EGFP. This detection system is expected to be a powerful tool in the field of biological processes (Shibasaki et al., 2003).

Yeast display library technology is a powerful tool for isolation and identification of proteins with unique or modified properties or selection of specific substances (including functional antibodies, enzymes, active protein sites, and chemical probes, *etc.*), and further identification in combination with fluorescence-activated cell sorting (FACS) (Jeong et al., 2019; Bacon et al., 2020; Banach et al., 2022; Fiebig et al., 2022; Lerma Romero et al., 2022). *S. cerevisiae* surface display system could be designed using inefficient ribosomal skipping, and the protein of interest could be simultaneously displayed on the cell surface and secreted out of the cell (Cruz-Teran et al., 2017). A Multiple Navigation of Antibody Structures (MINAS) method that combines CRISPR/Cas9-based traceable editing and fluorescence-activated cell sorting (FACS) of yeast-display libraries has been successfully designed to act on any region of the antibody, introducing hundreds of thousands of mutations and mapping the effect of these mutations on the desired phenotype (Oh et al., 2020). The optimization of the yeast surface display system is helpful in improving the application of the system in screening (Kajiwara et al., 2020).

In addition, cell surface display engineered *S. cerevisiae* encapsulated by biological materials can be used for protein purification. Taking advantage of the high affinity between *E. coli* vegetal E7 deoxyribonuclease (CL7) and its inhibitor immunoprotein 7 (IM7), calcium alginate beads encapsulated with *S. cerevisiae* cells displaying IM7 could be used as column filler materials to purify proteins fused with CL7 tags. Calcium alginate beads encapsulated with yeast cells displaying cellulosomes could act as a whole-cell catalyst to degrade cellulose substrates, which could be reused at least six times with very low activity reduction (Yin et al., 2022). This strategy of encapsulating cell surface displaying engineered bacteria will play an important role in pharmaceutical and bioengineering fields.

## 4 Current challenges and future prospects

Yeast cell surface display technology has developed rapidly in the past few decades, and different display systems have been designed. There are some bottlenecks in the development process of widely used *S. cerevisiae* cell surface display engineering: 1) Inefficient production of heterologous proteins leads to the lack of activity of engineered yeast strains; 2) with the increases in recycling times, the activity of engineered yeast may decrease more; 3) more attempts are needed in terms of improving the surface space utilization of yeast cells; 4) most of the current studies are mainly at the laboratory level, and further investigation are needed on the suitability of the constructed engineered strains for industrial mass production; 5) molecular and physiological knowledge on the tolerance of engineered yeast to many inhibitory compounds is limited.

To solve the above bottlenecks, future research can focus on the following aspects: 1) more novel anchoring proteins should be developed to extend the cell surface displayable sites and new gene editing systems combined with cell surface display should be designed to increase the copy number of target genes and simplify the process of plasmid transformation; 2) more biomaterials for encapsulating engineered yeasts should be developed to increase the times of reuses, maintain the activity of display proteins, and further expand the application areas. In addition, the system design to induce automatic flocculation of *S. cerevisiae* is also helpful to promote the yeast recovery; 3) constructing *S. cerevisiae* cell surface display systems by rational and systematic design, including the regulation of the location and ratio of multiple target proteins displayed on the cell surface and the way multifunctional scaffold proteins assembled on the cell surface. Exploring effective and versatile linkers is essential to improve the efficiency of cell surface display; 4) the combination of cell surface display technology, metabolic engineering and synthetic biology engineering is helpful to improve the ability of engineered yeast to utilize mixed substrates, reduce the production of by-products, and construct multifunctional “super yeast”; 5) to improve the tolerance of engineered strains to inhibitors or toxic substances in the fermentation process by means of strain mutagenesis, directed evolution, genetic engineering. The capability of engineered *S. cerevisiae* should be evaluated in a simulated industrial production environment.

## 5 Conclusion

In this paper, recent advances of cell-surface display engineering and the strategies to improve the display efficiency of *S. cerevisiae* are reviewed. In spite of the obvious requirement for increasing the amount of proteins that displayed on the cell surface and the efficient expression and secretion of target proteins, it is expected that more products using cell surface display will be commercialized in the future.
